# Process Development of a Generative Method for Partial and Controlled Integration of Active Substances into Open-Porous Matrix Structures

**DOI:** 10.3390/ma16216985

**Published:** 2023-10-31

**Authors:** Lena Burger, Achim Conzelmann, Sven Ulrich, Hadi Mozaffari-Jovein

**Affiliations:** 1 Institute of Materials Science and Engineering Tuttlingen (IWAT), Campus Tuttlingen, Furtwangen University, 78532 Tuttlingen, Germany; 2 Institute for Applied Materials-Applied Materials Physics (IAM-AWP), Karlsruhe Institute of Technology, 76131 Karlsruhe, Germany; 3 Department of Microsystems Engineering, University of Freiburg, Georges-Köhler-Allee 103, 79110 Freiburg, Germany

**Keywords:** drug delivery system, bioprinting, surface functionalisation, antibacterial agents, hydrogel, titanium matrix, porous matrix, contact angle, additive manufacturing

## Abstract

A special generative manufacturing (AM) process was developed for the partial integration of active ingredients into open-porous matrix structures. A mixture of a silver-containing solution as an antibacterial material with an alginate hydrogel as a carrier system was produced as the active ingredient. The AM process developed was used to introduce the active ingredient solution into an open-porous niobium containing a β-titanium matrix structure, thus creating a reproducible active ingredient delivery system. The matrix structure had already been produced in a separate AM process by means of selective laser melting (SLM). The main advantage of this process is the ability to control porosity with high precision. To determine optimal surface conditions for the integration of active ingredients into the matrix structure, different surface conditions of the titanium substrate were tested for their impact on wetting behaviour of a silver-containing hydrogel solution. The solution-substrate contact angle was measured and evaluated to determine the most favourable surface condition. To develop the generative manufacturing process, an FDM printer underwent modifications that permitted partial application of the drug solution to the structure in accordance with the bioprinting principle. The modified process enabled flexible control and programming of both the position and volume of the applied drug. Furthermore, the process was able to fill up to 95% of the titanium matrix body pore volume used. The customised application of drug carriers onto implants as a drug delivery system can be achieved via the developed process, providing an alternative to established methods like dip coating that lack this capability.

## 1. Introduction

Successful orthopedic implant systems are designed to restore the physical function of the affected joint or bone and to promote bone regeneration. Accordingly, they must have properties that mimic natural bone, i.e., adapted mechanical and biocompatibility behaviour.

In addition, the implants used should not release unwanted toxins or cause bone cells to be shielded from mechanical stress (stress shielding behaviour). An improved antimicrobial capacity of the implant, for example through the use of drug delivery systems, is intended to combat microbially induced local inflammation and promote the ability to regenerate bone [1,2,3].

A contemporary implant must fulfil all requirements objectively. The interlocking of modern techniques is necessary to reach such objectives. This paper addresses these challenges by combining two processes. The implant or the matrix structures were produced by using the process of selective laser melting (SLM) [4,5]. The body samples created using this methodology from a niobium-inclusive β-titanium base alloy with a specific porosity were functionalised by means of a modified bioprinting process [4,6].

Selective laser melting (SLM) aims to meet the requirements of personalised and more precisely fitting implants [6,7]. Patients ought to attain optimal freedom of movement while wearing the implant, thereby facilitating a more natural experience. SLM also provides a great deal of design flexibility and accuracy, even in detailed structures. Hence, it is feasible to tailor implants both functionally and individually to the patient’s specifications. The implants produced through the SLM process can accurately depict the glenoid’s anatomy and bone structure while also withstanding physiological stresses. In contrast, traditional manufacturing methods only allow for limited production of custom-fitted implants. With SLM, each component can be manufactured to have variable geometries and unique properties.

Introducing an open-pore surface structure could enhance the osseointegration of the implant, improving the connection between the implant and bone cells [8]. The spatial design of the porous support, the size and distribution of pores, and their accessibility for cells and for exchanging nutrients are all vital factors for cell growth and differentiation [9]. The distribution of open pores and the structural architecture of an implant are additional factors impacting its interaction with surrounding tissue. These structural elements may be arranged according to stochastic, fractal, or periodic principles, which significantly impact both intercellular coordination and mechanical properties of the inserted implants [10]. In addition to enhancing the contact area between the implant and bone tissue, open-porous structures present the opportunity to integrate pharmaceutical agents, which can improve the healing process of the patient. As well as the implant’s structure, the material used is paramount for hip implant development. Ti-6Al-4V is a medically approved alloy that is used for various medical products due to its high biocompatibility and excellent mechanical and chemical properties. The titanium-based alloy used in this study exhibits a lower modulus of elasticity compared to other Ti-based alloys. This is advantageous in promoting good ingrowth behaviour and thus preventing the stress shielding phenomenon and associated aseptic loosening. It is essential to closely match the selected alloys modulus of elasticity with that of the bone to achieve optimal results. Therefore, the use of β-titanium alloys containing niobium was preferred here, as their modulus of elasticity is better adapted to the properties of the human body.

In addition to optimising the product design through construction and manufacturing process, the behaviour of the implant can be improved by functionalising the surface. The product surface serves as the most important contact point between the artificial implant and the organ cells. Thus, the product properties, such as chemical, physical but also topological, play a decisive role [11].

Clinical studies have demonstrated that the implant used during human implantation is a primary risk factor for infection development [12]. The placement of foreign material in the body can increase the risk of infection by a factor of between 10,000 and 100,000, attributable to an increased bacterial presence responsible for infection [13,14]. Implant surfaces are a possible contributing factor to the heightened risk of infection. In addition to the heightened risk of infection, the insertion of a foreign body surface can also encourage the development of biofilms [15,16]. These biofilms shield the enclosed bacteria from the body’s immune system and from antibacterial agents [17,18]. Combating a biofilm or the resulting infection necessitates significantly larger doses of antibiotics or other medications due to inadequate vascularisation at the infection sites [19,20]. Alternatively, an anti-adhesive coating can be applied on the implant surface to prevent the formation of bacterial biofilm. Research on such coating systems has demonstrated their ability to limit contact between potential pathogen sites on the surface and many bacterial species, preventing adhesion [21]. Nevertheless, this approach also impairs the adhesion of host cells. Thus, surface modifications are unsuitable for implant systems that necessitate bone ingrowth. Alternative options to drug treatment comprise debridement, replacement arthroplasty, explantation of the implant, or amputation of the affected limb due to the infection [22,23,24].

Based on the Annual Report of the Arthroplasty Registry, 17,752 follow-up procedures were carried out on hips in 2021, with infection cited as the primary reason [25]. To avoid infections at the implant site and consequently reduce the need for risky follow-up interventions, the creation and manufacturing of bespoke implants for patients has grown in significance in recent times. These implants are not only better suited to the anatomy of the individual patient but also facilitate the targeted diffusion of active substances and optimal integration into the bone tissue.

Most methods for destroying bacteria are based on mechanisms that hinder cellular respiration, cell division or cell wall formation [26]. Many pharmaceutically active materials can activate the mechanisms mentioned, and they can be organic or inorganic. Silver, an antimicrobial metal, is currently the most widely used in biomedical applications. The activity of dissolved silver cations results in the impairment of bacterial cell membrane permeability and cell metabolism. Additionally, silver plays a role in the creation of reactive oxygen compounds that have the potential to affect prokaryotic cells [27]. Even so, there are concerns over the toxicity of silver ions and their use in coatings. Even at low levels, silver can harm surrounding cells and cause potentially harmful local accumulations [28]. Nonetheless, there are already techniques available that concentrate on developing silver coating technologies, which significantly reduce or completely remove its toxicity without impeding its antibacterial effect [29,30].

Various methods exist for introducing active substances into open-porous structures. At present, techniques for coating matrix material to enhance osseointegration and antibacterial effectiveness include dip-coating processes. In such processes, the matrix material is dipped into the coating solution and then removed by dripping. New technologies, such as bioprinting, facilitate the layer-by-layer production of tissue substitutes or synthetic organs through generative manufacturing. Bioprinting enables the recreation of cell distributions of tissues and organs precisely in a three-dimensional space [31]. The biomaterials used in this process are hydrogels that are formulated with active substances or cells. Characteristic features of bio-inks include printability, biocompatibility, degradation kinetics, and their resulting degradation products [32].

This study aimed to assess the feasibility of a process combining these features with regard to their process influences and fluctuations, as well as their inflation and functionalisation behaviour. For this purpose, specific open-porous samples, created via the SLM technique, were infused with a modified bioprinting process that utilised a silver-containing hydrogel mixture to later analyse the effectiveness of the process amalgamation.

## 2. Materials and Methods

### 2.1. Open-Porous Matrix Structures

**Figure 1:**
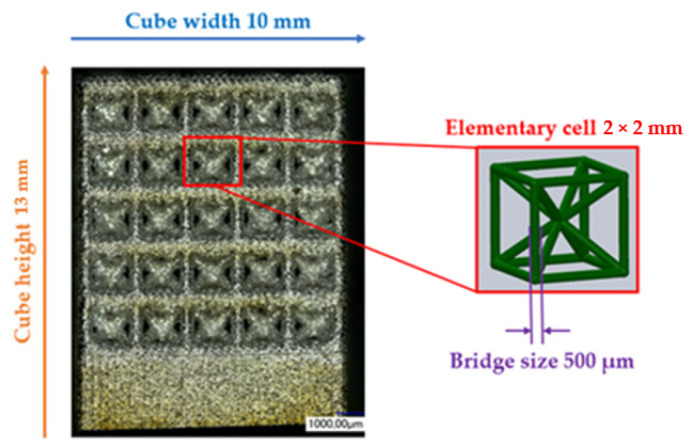
Dimensions of the BCC open-porous structures and their desired values.

**Figure 2:**
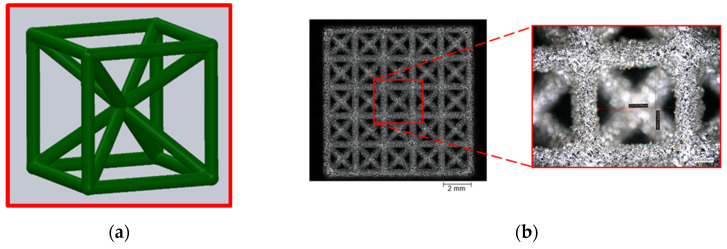
(**a**) Model used for the fabrication of the cubic space-centred unit cell; (**b**) top surface of a BCC matrix fabricated by SLM.

The matrices utilised for this study were fashioned from titanium-based alloys and manufactured using additive techniques. The powder bed-based selective laser melting (SLM) process allowed for the construction of intricate matrix structures as a result of its considerable design flexibility. Samples were generated in the Concept Laser Mlab while being attended to by a controlled argon inert-gas ambience. The structures were designed to guarantee bone ingrowth while ensuring stability in the load-bearing area. Technical term abbreviations are explained when first used. Figure 1 displays the anticipated values for the open-porous matrix structures. The functionalisation examinations in Figure 2 were carried out on structures with recurring body-centred cubic (BCC) elementary cells. After the SLM process, the samples underwent cleaning in an ultrasonic bath with ethanol in various stages to eliminate particles from the interior of the structures.

The hydrogel’s penetration behaviour into the pores was evaluated via the measurement of the contact angle θ with the matrix structure. To achieve this, SLM techniques were employed in the production of a compact material with varied surface properties.

### 2.2. Sample Preparation

To assess the impact of distinct surface textures on the performance of the drug delivery system, we previously analyzed the sample surface utilizing a profilometer and optical microscopy. The SURFCOM 1500SD-13 surface measurement system by Zeiss was employed for gauging the surface roughness of the specimens. In this measurement, we determined the arithmetic mean roughness value R_a_ and the arithmetic mean value of the individual roughness depths R_z_ for the three surfaces based on the guidelines of DIN EN ISO 4287 and ASME B46.1. A measuring distance of 8000 mm and a measuring speed of 0.300 mm/s were chosen for the roughness measurement at room temperature. The selection of the measurement parameters relied on the choice of the measurement section for aperiodic profiles according to the standard DIN EN ISO 4288. The measurement was repeated five times per condition, and the resulting data were used to calculate the arithmetic mean and standard deviation for the average roughness R_a_ and roughness depth R_z_ parameters.

The Keyence digital microscopes VHX-1000 (magnification 50–200×) and VHX-5000 (magnification 100–1000×) were primarily used for surface characterisation and to measure the BCC structure parameters indicated in Figure 1. All findings are reported herein in an objective and impartial manner. The structural parameters were measured 30 times, and the mean value and standard deviation were calculated. Static contact angle measurements were conducted on samples exhibiting three distinct surface states. The specimens included three conditions: the untreated state immediately after manufacturing (as-built), a surface that had been ground, and a surface that had been polished. The preparation of the specimens was carried out in accordance with the parameters specified in Table 1.

### 2.3. Preparation of the Drug Delivery Solution

To investigate the interaction behaviour of a bio-ink on various titanium surfaces, a sodium alginate hydrogel was utilized. In order to manage the infiltration of the hydrogel into the matrix, a cross-linking solution based on calcium chloride was added to the hydrogel solution. These cross-linking solutions were expected to offer optimal control over the quantity of biopolymer introduced. The experimental solutions’ compositions are presented in Table 2.

The sodium alginate solution was obtained from the powdered form, as indicated in Table 2. Deionised water containing 100 ppm colloidal silver was used to prepare the solution. It is crucial to note that the liquid component must not contain any divalent ions during the production process to avoid sedimentation. The two components were mixed and continuously stirred at 60 °C until a homogeneous mixture was formed; then, 0.11 g of calcium chloride was weighed precisely on a balance and dissolved in 10 mL of functiona water to create the calcium chloride solution.

To assess the infiltration and wetting behaviour of the silver-containing alginate solution, as well as its stability after cross-linking, contact angle measurements were employed as an evaluation method. Therefore, bulk material (refer to Table 1) was used as a representative for the measurements, as it was very problematic to measure the contact angle on or within the structure due to its high complexity. The infiltration behaviour of the biopolymer also depends on the porosity generated in the substrate material resulting from the SLM process. Thus, the volume and density of the structures were assessed before and after functionalisation of the BCC specimens to consider this factor. The subsequent sections detail the methods utilised.

### 2.4. Measurement of the Static Contact Angle θ

As part of the wetting behaviour assessment, contact angle measurements were taken on the previously mentioned bulk material of the β-titanium-based alloy containing niobium.

To achieve this, a measuring setup was employed where the specimen was positioned in a chamber located in front of a light source with a frosted glass plate. Fluids were dosed through the top chamber opening using syringes. An endoscope (KARL STORZ), which could be adjusted to the height of the relevant sample, was utilized to capture images. The gathered data were subsequently transferred to an associated monitor or data carrier for evaluation. Herein, the images produced were assessed based on the angular ratio between the bulk material and the generated solution droplet to derive the corresponding static contact angles θ without the use of software only by visual assessment. The contact angle was measured three times for each drop and an average value was calculated.

### 2.5. Measurement of Volume and Density

In relation to the functionalisation of the titanium structures employed, the density was determined using Archimedes’ principle. The resulting data were subsequently function to determine the structure filling. The method employed to determine density based on Archimedes’ principle is founded on buoyancy. An electrical precision balance, which was adapted as a hydrostatic balance from Sartorius, was employed for this purpose. In order to determine density, the mass of the sample was initially measured in air. The mass in ethanol was measured to determine the density and volume of the samples. These values formed the foundation for ascertaining the density and volume of the samples. The measurement process was done five times to calculate the arithmetic mean for all samples.

### 2.6. Determination of the Structural Filling

A gravimetric approach was chosen to evaluate the percentage of structure filling or the free residual volume in the structure. For this purpose, the structure was weighed before functionalisation and its initial mass m_1_ was determined. After functionalisation, the sample was weighed again to determine the total mass m_2_ after functionalisation. After subtracting the masses, the difference is the mass of the alginate-containing solution m_A_. Together with the density ρ_A_ of the respective alginate solution concentration, the solution volume V_A_ could then be determined. To determine the free volume V_Pores_ of a BCC structure, the measured volume of the BCC structure is subtracted from the volume of a cube of the same size (10 mm × 10 mm × 13 mm) made of bulk material. The percentage structure filling is the ratio of the volume V_A_ to V_Pores_ multiplied by a factor of 100.

### 2.7. Functionalisation Process

In order to be able to introduce the selected carrier in combination with an active ingredient into the structures, the FDM printer had to be modified in several steps into a functionalisation system. This subsection deals with the necessary modifications and their implementation.

The first step was to construct an enclosure that provided a constant temperature field for processing the alginate-containing solution.

**Figure 3:**
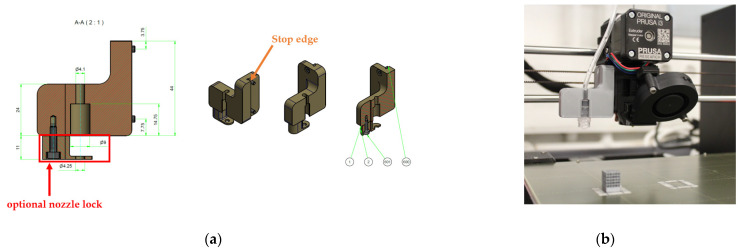
Structure of the printed holder with Luer lock connection, (**a**) sectional drawing of the nozzle holder, and (**b**) assembled state.

The second step in the modification of the printer was the development of a holder for the functionalisation nozzle. A section of the parts drawing for the holder is shown in Figure 3a on the left. The bracket could be easily attached to the fan of the extruder unit using two M3 × 12 screws. In addition to the fixing to the fan housing, a stop edge (orange arrow) was included in the design to ensure that it was flush with the fan housing when correctly fitted. If the functionalisation nozzle, which was designed as a Luer lock connection, was not fully seated in the holder, it could be additionally secured using a clamping bracket and an M3 × 8 screw (red frame). The Luer lock holder mounted on the printer is shown on the right in Figure 3b.

In addition to the design and manufacture of the holder for the functionalization nozzle, a pump system was required to deliver the drug delivery solution. As the pump had to deliver small volumes, the syringe pump (Perfusor^®^ Space) from B. Braun SE was chosen. In addition to setting the flow rate in various units, the pump also offered the option of specifying a total volume to be delivered or a delivery time. Syringes (original Perfusor^®^ syringes) with a total volume of 20 mL were used as reservoirs for the silver-containing alginate solution to be pumped, specifically designed for use in the syringe pump mentioned above.

An infusion tube, called a Heidelberg extension, with a Luer lock connector on one end and a cap on the other end, was used to connect the setup system to the syringes.

**Figure 4:**
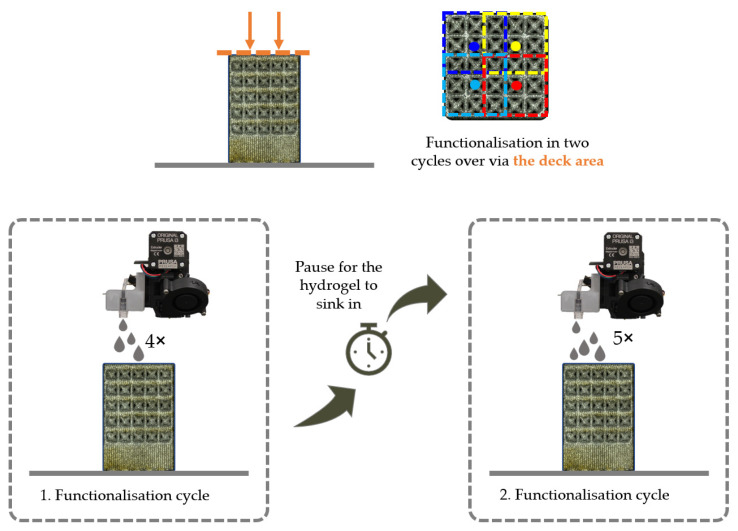
Sequence of the filling process over the top surface of the BCC structure.

The matrix structure was filled exclusively from the surface of the manufactured component. This was done in two cycles with a pause between the first and second cycle to allow the alginate solution to penetrate the structure by gravity. The process flow for this variant is shown in Figure 4 and described below.

For the test run, a programme was created that included the calibration of the print bed, the start position of the nozzle (G1 X96.000 Y160.000), which was in the centre of the left side of the printing plate, and the speed at which the nozzle moved (G1 F1200.000).

After these presettings, the nozzle remained in the approached start position for approximately 106 s to build up the pressure stage. If the pressure buildup time was set to 99 s, the nozzle would remain in this position for a further seven seconds and then move to the new position just before the drop broke. During the pressure buildup, a plastic Petri dish was placed under the nozzle to collect the outflowing solution. During this pause, the BCC structure was placed on the print bed. The position was measured and marked with the g-code. After the pause, the nozzle moved with the command G1 to the position of the blue point (X101,000 Y129,000). This position was 5 mm further to the right and 31 mm below the start position described before. The nozzle remained at this position for 13 s.

This procedure was repeated in the same way for the remaining groups of unit cells (see blue, yellow, and red squares in Figure 4). When all the groups had been wetted with a drop of the alginate-containing solution, the nozzle was moved to a pause position next to the structure for 100 s. The cycle just described was then repeated a second time. In the second cycle, an additional drop was added to the yellow area to improve the filling of the structure. At the end of the second cycle, the nozzle returned to the home position and the pump could be switched off. Before the structure could be removed from the housing, it was necessary to wait again until the alginate solution had completely penetrated the structure and no more alginate solution was visible on the surface of the deck.

## 3. Results

### 3.1. Surface Characterisation

**Figure 5:**
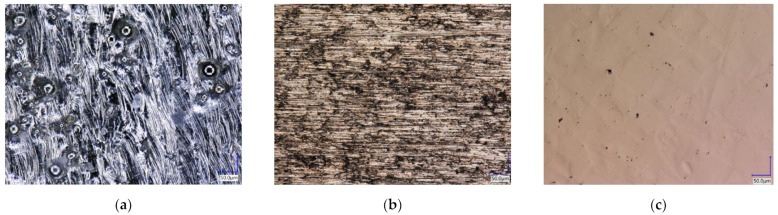
Surface conditions of the additive manufactured titanium-based alloy (**a**) as-built (500×); (**b**) ground (500×); (**c**) ground and polished (500×).

The light microscope images of the three surface conditions are shown in Figure 5. The surface quality was used to classify the results of the contact angle measurements. The behaviour of the biopolymer and crosslinker was not only related to the surface roughness that could be measured, but also to the characteristics of the topography. The as-delivered sample (Figure 5a), which was not treated after the SLM process, had spherical silver shiny inclusions of different sizes on the surface. These inclusions were located in process-induced crater-like depressions on the surface. The surface was also silver lustrous and had an irregular surface topography. The ground surface showed a uniform, periodic pattern on the surface, mainly characterised by grooves of different sizes resulting from the grinding process (see Figure 5b). The polished sample was characterised by small dark pores on the surface and faintly visible smaller areas arranged in a square (see Figure 5c).

The three surface conditions of the samples were characterised using a profilometer as described in Section 2.2. The average roughness R_a_ and the average roughness depth R_z_ were measured (see Table 3). The light microscopic results (see Figure 5) are also reflected in the roughness parameters. For the unground surface, the average R_a_ was 8.34 µm and R_z_ was 10.53 µm. For the ground surface, the characteristic roughness values according to the SEM images decreased to 0.323 µm for R_a_ and 2.332 µm for R_z_. The ground and polished surface had a mean roughness of 0.098 µm and a mean roughness depth R_z_ of 1.548 µm.

### 3.2. Geometrical Characterisation of the Matrix Structures

The matrix structures produced were also characterised under the optical microscope after cleaning. In addition to the ridges, the width and height of the cube and the size of the deliberately created pores were documented.

The geometric characterisation of the samples was used to assess the quality of the SLM process. Figure 1 defines the target dimensions for the SLM process. After fabrication and cleaning of the structures, these dimensions must be checked to document geometric deviations such as warpage or process-related satellite adhesion. Table 4 below shows the mean values and deviations for the relevant structural parameters. The measured values for bridge size and elementary cell width were above the specified target values. This may have been caused by adhering powder that had not been removed. The height and width of the cube, on the other hand, indicated shrinkage of the sample.

### 3.3. Characterisation of the Drug Delivery Solution

Before the drug delivery solution could be used, its wetting behaviour on different surfaces and its flow behaviour at different concentrations were determined.

For the three different surface conditions, five concentrations of the hydrogel were studied to determine the wetting behaviour on different surface topographies as a function of viscosity. Three photographs were taken of each droplet and the contact angles for each state and concentration were determined and averaged. In the following Table 5, Table 6 and Table 7, a drop is shown as a representative example. First the drop of alginate solution was photographed after it had been applied to the surface. Then, a picture was taken after the addition of the calcium chloride solution. After a subsequent cross-linking time of ten minutes, the third state was documented. The curing times were based on research in the literature and information from the manufacturer. The results of the contact angles thus determined for all surface states are shown in the following three tables (see Table 5, Table 6 and Table 7). For all three surface states, a strong change in viscosity was observed in the third state after the addition of the cross-linking solution.

The behaviour of the hydrogel mass changed from liquid to gel-like consistency to a solid state. After the addition of the cross-linking solution, the alginate compound formed spherical gels at concentrations of 2.5 wt.% and 3 wt.%, which no longer exhibited adhesion to the bulk material.

**Figure 6:**
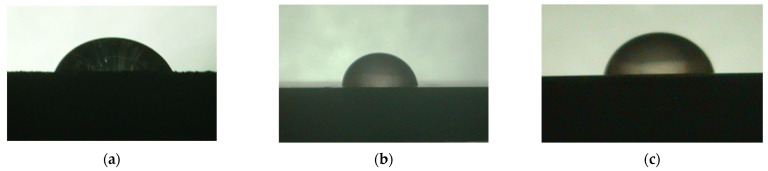
State 1 of the 2 wt.% alginate solution on the (**a**) as-built surface, (**b**) the ground surface and (**c**) the polished surface.

The following detailed explanations refer to the 2 wt.% alginate solution, as this gave the best results and was used for further testing. Concentrations above the 2 wt.% limit led to a sharp increase in viscosity (see Figure 6). This means that extrusion from the die was no longer possible in the subsequent functionalisation process. Concentrations below 2 wt.% had too low a viscosity, which means that the drug delivery solution did not have a suitable viscosity and could leak out of the structural cells.

**Figure 7:**
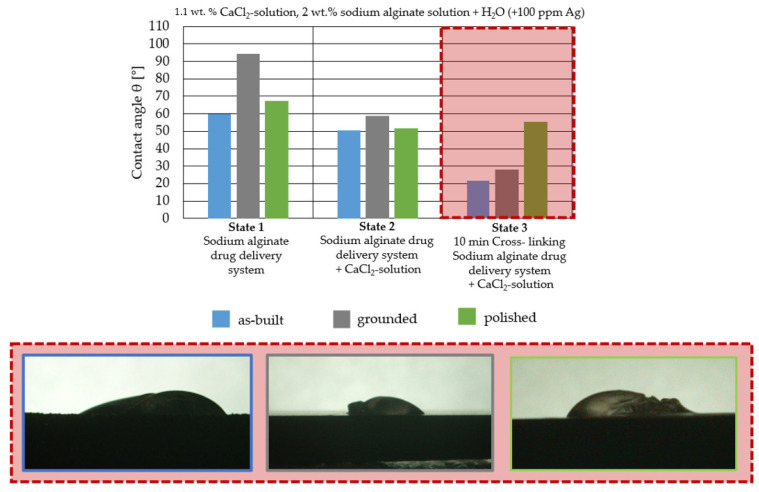
Change in contact angle between states 1 and 3 depending on surface condition.

Both the as-built surface and the polished surface had a larger wetting area than the drop on the ground surface. Figure 7 shows the measured contact angles for both the state just considered and the other two states as a function of the three surfaces. The smaller contact area on the ground surface resulted in a contact angle of 94°. The alginate solution showed contact angles of 60° and 67° on the unpolished and polished surfaces. In condition 2, after the addition of the calcium chloride solution, a decrease in the wetting angles could be observed for all three surfaces. These were now 50.5° on the as-built sample, 58.5° on the ground sample, and 51.5° on the polished sample. As in condition 1, the ground surface showed the largest contact angles. The alginate-containing solution showed a 1° larger contact angle on the polished surface than on the as-built sample. Due to the strong cross-linking by the CaCl_2_ solution, a solid mass was formed in the third state, which could no longer be considered as a liquid. In this case, contact angle analysis could only be used for the first two states. For completeness, all states are shown in Figure 7.

The contact angles for the third state were measured on the drop side, which showed a clear contour and was not characterised by discontinuities (see Figure 7, green frame). There were clear decreases for the ground sample, while the polished sample increased to values above the second state. On the ground surface, the contact angle again decreased significantly to 21.5° compared to the previous state.

As already mentioned, the drop contours in the third state were partly irregular and therefore not clearly visible. The viscosity of the alginate-containing mass initially applied also changes over the course of the three states. The alginate solution had a higher viscosity than the cross-linking solution. By combining the two solutions in the second state, the viscosity increased again significantly and a solid elastic mass was formed, which was no longer comparable to a liquid due to its gel-like appearance.

### 3.4. Assessment of the Quality of the Functionalisation

A qualitative and a quantitative method were used to evaluate the quality of the functionalisation in terms of structure filling of the silver-containing alginate solution.

**Figure 8:**
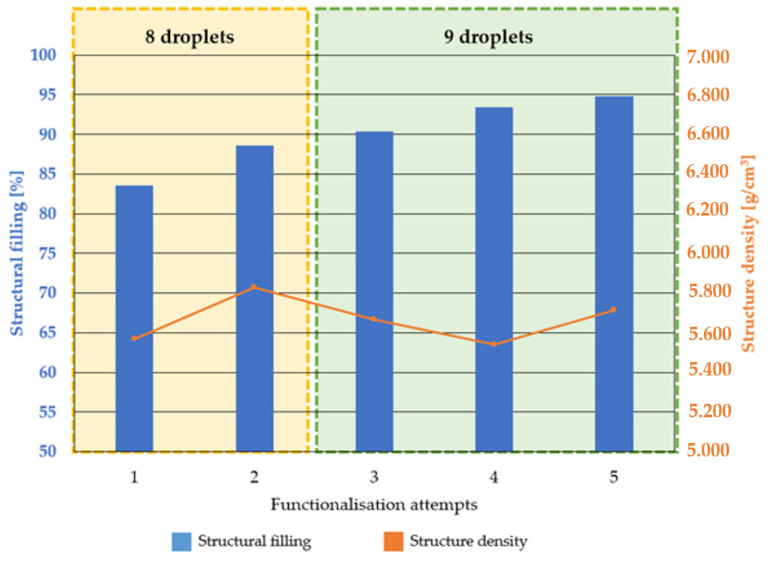
Structure filling and initial structure density for the functionalisation tests.

Functionalisation experiments three to five in Figure 8 (green) were carried out with a total of nine drops, while experiments one and two, framed in yellow, were carried out with a total of eight drops over the two functionalisation cycles. A maximum of 95% structural filling was achieved with nine drops. In addition to the structure filling, the density of the individual BCC structures is shown in the graph. There was a tendency that structures with a lower initial density before functionalisation showed a higher structure filling for a constant number of drops. As an example, the samples with a constant number of eight drops are considered. In the first test, a BCC structure was used, which had few adhesions and residual powder in the structure due to the process or cleaning. In addition to the structural aspects, it was therefore possible to draw conclusions about the quality of the cleaning process and the presence of SLM process-related satellite particles. The low density resulted in a high free pore volume. The eight filled droplets resulted in a low structural filling. Sample 2 is considered in comparison. In it, the mass and therefore the density increased after the SLM process and cleaning. There was less free pore volume in the structure, resulting in an apparently higher structure filling with the same number of drops. The tendencies described above could also be seen in experiments three to five with nine drops.

**Figure 9:**
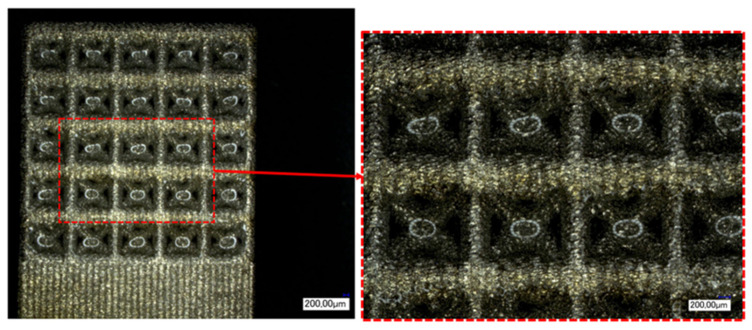
Light microscope image of the functionalised surface (20×) and detailed image of the elementary cells (50×).

The light microscopy images were used to assess the distribution of the hydrogel in the outer part of the structure. However, it was not possible to visualise the centre of the structure. On the one hand, the contrast of the organic hydrogel was not sufficient for computed tomography, and on the other hand, a light microscope cannot image the depth of the structure. Figure 9 shows the side surface of the first functionalisation experiment.

Due to the high transparency of the alginate-containing solution, it could not be made directly visible under the light microscope. However, the reflections of the ring light could be seen in the elementary cells filled with the alginate solution (see Figure 9). Based on this indirect view, it was possible to see that all cells down to the lower rows had been filled by the functionalisation of the top surface and the subsequent seepage of the solution into the structure. It could also be seen that the outer surface of the bulk material under the last row, which served to stabilise the structures, was not wetted by the hydrogel. Consequently, no alginate-containing solution leaked from the structure during the process.

## 4. Discussion

The aim of this research was to develop a process, based on generative manufacturing, that enabled the controllable functionalisation of a surface or structure with a drug delivery function.

There are two main reasons for developing such a novel functionalisation process. As mentioned above, postoperative infections are one of the most serious risks following joint replacement with a load-bearing implant. These are usually associated with complex revision surgery. Antibiotic treatment is often used to manage these risks, but this is only partially effective because the implants are usually located in areas of the body with poor blood supply. Such infections should therefore be avoided as far as possible. Another aspect is the established and available methods and processes that enable us to apply such functional substances to the surface of the implant in a controlled manner and thus develop customised drug delivery systems.

One example is dip coating, where implants are often manually dipped into the coating system and then drained. The process is highly user-dependent and only allows the coating of the entire surface of the component; variations in the amount of coating can occur. In addition, the new process allows for the partial and flexible introduction of substances into the implant, even in a gradient state.

To overcome the problem of undefined application volumes in the dip-coating process, the volumes in the new system were delivered by a syringe pump system, which is commonly used for infusion administration in hospitals and clinics for patient care. As described, the pump allows the volumes introduced into the structures to be precisely defined by defining variable parameters such as flow rate. In addition to the precise definition of the flow rate, another advantage of the pump is that it significantly increases material utilisation, as only the required material is used.

In order to functionalise the BCC structures used, or to functionalise them via the deck of the BCC structures, it was necessary to find a way of moving the extruder head with the attached functionalisation nozzle in such a way that a controllable and targeted delivery of volumes was possible. The use of the structures in a BCC constellation was based on the evaluation of different matrix structures using the Maxwell stability criteria. Here, the BCC structure showed the highest mechanical stability with respect to the existing stiff bars and nodes. The g-code was used for this purpose, allowing the printer to perform the desired movements in a programmable manner.

The g-code was limited to the movements of the extruder head and pressure bed and did not provide a communication interface with the pump. At the current stage of development, the use of the pump requires manual operation. If the time for starting the pump is exceeded due to operator error, insufficient structure filling may occur. As this possible error influence could be detected at an early stage of development, it was countered in two ways. On the one hand, to minimise or eliminate the possibility of operator error, the process was annotated so that it could be monitored, as each step in the programme was clearly marked. On the other hand, the programme was timed to the pump so that small delays in starting the pump could be compensated for by a correspondingly longer pause time. An example of this was starting the pump before the first cycle. The measured times for pressure buildup in the pump indicated a time of 99 s. However, the programme allowed for a pause of 106 s, further ensuring that the nozzle moved to the first position just before the drop broke. This also ensured that if the pump was started at a later time, it would not place too few drops on the surface of the structure. By using the pump system in conjunction with the g-codes, uncontrolled coating processes were virtually eliminated. Further optimisation can be achieved by fine-tuning the interface so that the coordination between the pump and the movement of the printer is completely eliminated.

The nozzle, its delivery system, and mounting change a key aspect of the coating process. In the dip-coating process, the component is immersed in the coating system; here, this relationship was reversed and the coating system was deposited at controllable locations on the component. This reversal increased the control and efficiency of the process by reducing the amount of coating required, as only the amount of coating needed was applied. In addition, dip-coating processes can carry contamination from one component to another, as the baths are not exchanged between each part to be coated. Bath carry-over is not possible with this new process.

Once the problem of poor control of the coating process had been overcome by the development of the functionalisation system, a suitable drug delivery solution was defined to prevent postoperative infections on and around the implant.

As it is not possible to reliably assess the wetting behaviour after insertion into the structure, these investigations were carried out on bulk materials. The contact angle measurements showed that the wetting behaviour of the alginate-containing solution was best on the polished or “as-built” surface due to the low contact angle and thus larger wetting area. The polished surface showed a contact angle of 94°, especially in the first state. A contact angle greater than 90° was described as non-wetting or hydrophobic and thus represented a very low wetting of the surface. However, since a very good wetting of the webs was desired for the application of the hydrogel in the structure, the ground surface already showed an insufficient wetting behaviour in this respect. Another phenomenon seen on the bottom surface was particularly evident when looking at the second and third states.

While the application of the cross-linking solution to the polished and untreated surfaces resulted in pure solidification of the gel, the application of the solution to the grounded surface resulted in complete moulding and consequent detachment of the hydrogel from the surface. A possible explanation for this behaviour lies in the surface structure of the titanium surface. The ground surface consisted of a pattern of deep grooves created by the grinding process. These grooves may have acted as channels for the calcium chloride solution applied. This effect can also be seen on honed components. Fine grooves or honing lines are deliberately created in the surface, which can be used to allow fluids to flow [32].

The applied hydrogel was washed out by the cross-linking solution due to the presence of channels, resulting in all-round cross-linking of the gel, whereas the as-built surface, for example, did not allow such undercutting due to its aperiodic crater structure.

In addition to these observations, it could also be seen that the contact angle decreased from state 1 to state 2, regardless of the surface. A decrease in the contact angle could also be observed for the third state. However, it was noticeable that the drop shapes were very irregular when looking at the last state. The contact angle for the polished surface also increased significantly compared to the previous states. This can be explained by the applicability of contact angle measurement to liquids. The contact angle describes the angle between a solid surface and the liquid applied to it; in this case, the alginate solution. As shown in Section 3, a change in viscosity of the alginate solution could be observed due to the cross-linking solution. Therefore, this approach could not be used for the third state as the gel-like elastic mass was no longer a liquid. On the basis of the results described above, consideration of the results was limited to the first and second states. It is clear that the untreated and polished samples produced the best possible wetting, which was further improved by the cross-linking solution.

It should be noted that all measurements were static contact angle measurements. These only provide a molecular record of the current state and therefore cannot provide a dynamic record over time. The performance of further dynamic contact angle measurements could better illustrate the behaviour of the hydrogel in relation to sinking under gravity and should therefore be considered in future measurements. In addition to the measurements on different surfaces, different concentrations of alginate solutions were also investigated. While the solutions above 2.5 wt.% alginate showed a very viscous behaviour, the cross-linking of the solution with 1 wt.% alginate was hardly possible. Tests in combination with the pumping system showed that the 2 wt.% solution could be processed best compared to the other concentrations, as all other solutions led to a strong pressure increase in the dosing system due to their viscous state, which resulted in an error message.

The structures were functionalised with the alginate-containing solution (2 wt.% by weight) in two cycles. The hydrogel seeped through the surface by gravity. By functionalising with nine drops, a structure filling of more than 90% could be achieved. It can therefore be assumed that almost all of the open-pore volume in the structure was filled with the drug delivery solution. It could also be shown that additional cross-linking with the calcium chloride solution after functionalisation reduced the structure filling by about 70% in a preliminary test. One explanation for the reduced structure filling due to cross-linking could be that the mass withdrew into the interior of the structure during cross-linking and was therefore no longer visible at the edges. Another possible explanation could be that the immersion of the structures in the cross-linking solution washed out some of the solution containing the alginate. However, this does not necessarily mean that the cross-linking solution cannot be applied. It is possible to create partial or gradual coatings. This could be done by using several alginate-based hydrogels; for example, with different active ingredients or active ingredient concentrations. For example, an implant could be functionalised at a specific site, this site could then be cross-linked and the cross-linked area could be expanded with another drug component. Other active ingredients with antibacterial activity, such as copper, could also be considered, particularly as the efficacy of silver has not yet been evaluated [33,34]. In addition to the active ingredient, it is also possible to vary the carrier substance.

## 5. Conclusions

In summary, the objective of developing a process based on generative manufacturing for the targeted and controllable functionalisation of medical devices was achieved.

For this purpose:

- An FDM printer was converted into a functionalisation system in four steps.
- The BCC structures as well as the wetting and flow behaviour of the sodium alginate used as hydrogel for the drug delivery solution was characterised.
- It is clear that the alginate-containing solution led to the best possible wetting on the as-built and polished surfaces.
- However, this was only true for the first two states, as the solidification of the compound made it impossible to evaluate the contact angle in the third state.
- It was also shown that the 2 wt.% sodium alginate solution had a favourable flow behaviour for processing with the syringe pump.
- It was shown that with nine drops for the two cycles, the filling of the structure increased to more than 90%. These tests showed that, in addition to the process setup, the variations of the SLM process need to be taken into account. For future test series, the aim is to optimise the process in terms of cleaning the structures in order to achieve more consistent results in terms of free pore volume prior to functionalisation.

## Figures and Tables

**Table 1:** Preparation parameters for surface characterisation.

Sample	Preparation
sample 1	as-built
sample 2	grounded to grain size: 600 µm
sample 3	grounded and polished, grain size: 2500 µm; 1 µm; 0.06 µm

**Table 2:** Concentrations of the used sodium alginate and calcium chloride.

Solid Powder	Solvent	Concentration of the Solution [wt.%]
Sodium alginate	Deionised water with 100 ppm colloidal silver	1 2 2.5 3 5
Calcium chloride (CaCl_2_)	Deionised water	1.1

**Table 3:** Titanium base alloy roughness Ra and Rz for different surface conditions.

Roughness Characteristics *	As-Built	Grounded	Polished
Average roughness R_a_ [µm]	8.34 ± 0.2	0.323 ± 0.02	0.098 ± 0.01
Roughness depth R_z_ [µm]	10.53 ± 0.3	2.332 ± 0.18	1.548 ± 1.14

* Measuring distance: 8.000 mm | Measuring speed: 0.300 mm/s | Measured on bulk material.

**Table 4:** Structural parameters of the titanium base alloy matrix structures.

BCC Structure Parameters	Desired Values	Measured Values
Bridge size (µm]	500	517.82 ± 25
Elementary cell width (µm]	2000	2350.13 ± 27
Cube width (µm]	10,000	9754 ± 20
Cube height (µm]	13,000	12.725 ± 21

**Table 5:** Images of the wetting behaviour of the drug delivery solution on the as-built surface and static contact angle in °.

Concentration of the Drug Delivery Solution (%)	State 1	State 2	State 3
1	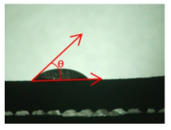	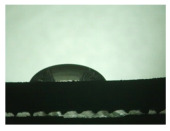	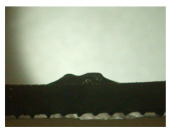
Contact angle (°)	47 ± 1	57 ± 2	24 ± 3
2	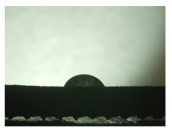	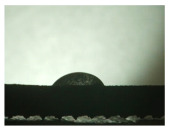	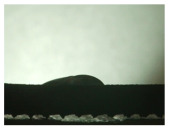
Contact angle (°)	60 ± 2	50.5 ± 4	21.5 ± 11
2.5	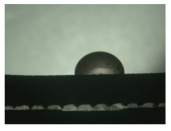	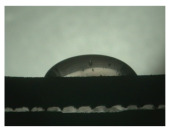	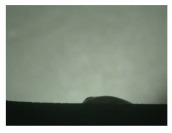
Contact angle (°)	85 ± 2	64 ± 6	53 ± 5
3	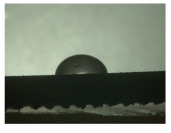	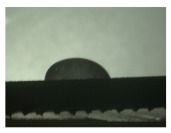	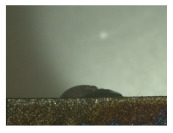
Contact angle (°)	84 ± 3	73 ± 10	47 ± 18
5	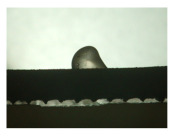	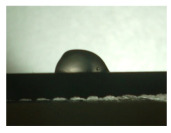	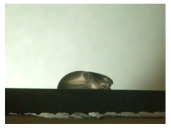
Contact angle (°)	83 ± 25	32 ± 1	55 ± 2

**Table 6:** Images of the wetting behaviour of the drug delivery solution on the grounded surface and static contact angle in °.

Concentration of the Drug Delivery Solution (%)	State 1	State 2	State 3
1	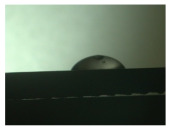	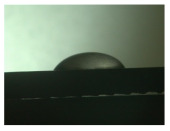	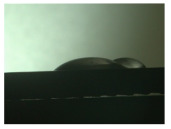
Contact angle (°)	51 ± 6	51 ± 6	30 ± 5
2	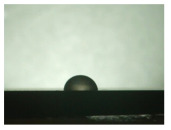	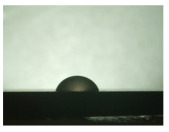	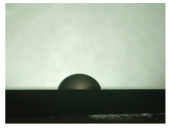
Contact angle (°)	94 ± 6	58.5 ± 2	28 ± 8
2.5	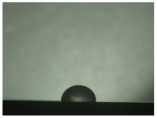	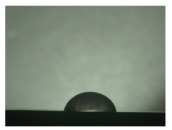	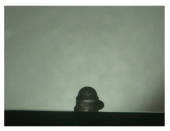
Contact angle (°)	82 ± 1	36 ± 19	57 ± 1
3	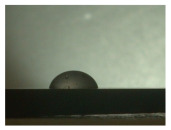	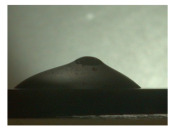	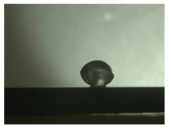
Contact angle (°)	76 ± 1	40 ± 2	75 ± 8
5	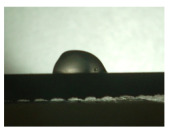	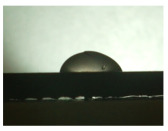	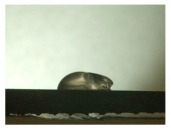
Contact angle (°)	73 ± 11	70 ± 6	62 ± 16

**Table 7:** Images of the wetting behaviour of the drug delivery solution on the polished surface and static contact angle in °.

Concentration of the Drug Delivery Solution (%)	State 1	State 2	State 3
1	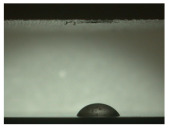	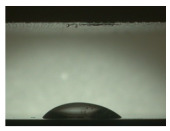	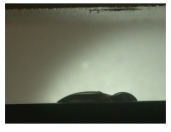
Contact angle (°)	55 ± 1	42 ± 4	43 ± 10
2	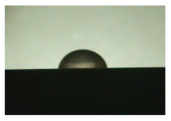	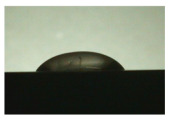	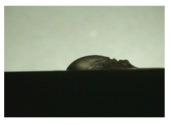
Contact angle (°)	67 ± 7	51.5 ± 2	55 ± 14
2.5	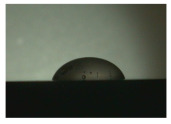	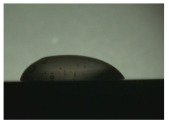	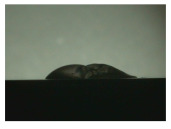
Contact angle (°)	70 ± 1	65 ± 2	45 ± 13
3	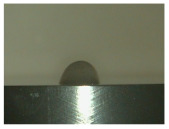	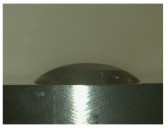	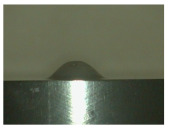
Contact angle (°)	77 ± 2	47 ± 5	34 ± 2
5	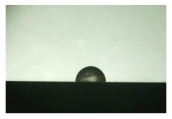	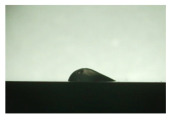	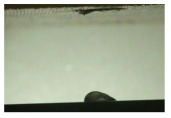
Contact angle (°)	76 ± 4	63 ± 27	46 ± 23

## Data Availability

The data presented in this study are available on request from the corresponding author.
